# Evaluation of Methionine Content in a High-Fat and Choline-Deficient Diet on Body Weight Gain and the Development of Non-Alcoholic Steatohepatitis in Mice

**DOI:** 10.1371/journal.pone.0164191

**Published:** 2016-10-10

**Authors:** Tsuyoshi Chiba, Sachina Suzuki, Yoko Sato, Tatsuki Itoh, Keizo Umegaki

**Affiliations:** 1 Information Center, National Institute of Health and Nutrition, National Institutes of Biomedical Innovation, Health and Nutrition, Shinjuku-ku, Tokyo, Japan; 2 Department of Food Science and Nutrition, Kinki University Faculty of Agriculture, Nakamachi, Nara, Japan; University of Catania, ITALY

## Abstract

**Aim:**

Non-alcoholic steatohepatitis (NASH) is a globally recognized liver disease. A methionine- and choline-deficient diet is used to induce NASH in mice; however, this diet also causes severe body weight loss. To resolve this issue, we examined the effects of methionine content in a high-fat and choline-deficient (HFCD) diet on body weight and the development of NASH in mice.

**Methods:**

C57BL/6J mice (male, 10 weeks of age) were fed an L-amino acid rodent (control) diet, high-fat (HF) diet, or HFCD diet containing various amounts of methionine (0.1–0.6% (w/w)) for 12 weeks. Plasma lipid levels, hepatic lipid content and inflammatory marker gene expression were measured, and a pathological analysis was conducted to evaluate NASH.

**Results:**

The 0.1% methionine in HFCD diet suppressed body weight gain, which was lower than that with control diet. On the other hand, the 0.2% methionine in HFCD diet yielded similar body weight gains as the control diet, while more than 0.4% methionine showed the same body weight gains as the HF diet. Liver weights and hepatic lipid contents were the greatest with 0.1% methionine and decreased in a methionine dose-dependent manner. Pathological analysis, NAFLD activity scores and gene expression levels in the liver revealed that 0.1% and 0.2% methionine for 12 weeks induced NASH, whereas 0.4% and 0.6% methionine attenuated the induction of NASH by HFCD diet. However, the 0.2% methionine in HFCD diet did not induce insulin resistance, despite the body weight gain.

**Conclusions:**

The 0.2% methionine in HFCD diet for 12 weeks was able to induce NASH without weight loss.

## Introduction

Non-alcoholic fatty liver disease (NAFLD) and the more progressive non-alcoholic steatohepatitis (NASH) have recently become recognized liver diseases in developed countries [1]. The prevalence of NAFLD has increased not only in Western countries, but also in Asia [2] and Japan [3, 4]. In addition, 10–15% of NAFLD patients have been diagnosed with NASH [5], which may progress from steatosis and fibrosis to cirrhosis and hepatocellular carcinoma [6, 7]. NASH is characterized by the liver biopsy findings observed in alcoholic hepatitis, in addition to elevated plasma indicators of hepatic damage and lipid accumulation in the liver with NAFLD. In humans, NASH is associated with obesity and metabolic syndrome, such as insulin resistance, diabetes mellitus and dyslipidemia [8, 9]. There is currently no established pharmacotherapy for NASH; therefore, animal studies are currently ongoing. However, even though several animal models of NASH have been reported [10, 11], an optimal animal model of human NASH does not exist.

A methionine and choline-deficient (MCD) diet produces a more progressive liver pathology characterized by the development of steatosis with inflammation and fibrosis in rodent models within a short time frame. This diet may induce steatosis and inflammation within 4 weeks. However, mice or rats fed MCD diet exhibit severe weight loss (due to a vastly lower caloric intake) and do not become insulin resistant [12, 13]. Most humans with NASH are obese and insulin resistant, and this is an important difference between MCD diet-induced NASH models and human NASH. A Western diet (45% energy from fat, 0.2% cholesterol, plus drinking water supplemented with fructose and glucose) is also used to induce NASH in mice. This diet induces obesity and insulin resistance, similar to that in humans. However, it has also been shown to induce less severe NASH than the MCD diet, even though mice were fed this diet for twice the duration of the MCD diet [14]. A high-fat MCD diet has also been used to induce NASH in mice. However, mice fed this diet failed to gain weight, similar to the MCD diet, even though the diet contained 40% (w/w) fat [15]. On the other hand, *db/db* mice fed MCD diet for 4 weeks showed insulin resistance, obese phenotype and greater body weight than mice fed a control diet. However, body weight was lower when compared to that before MCD feeding [16]. This suggests that the MCD diet suppressed body weight gain even in *db/db* mice.

Recent studies demonstrated that a high-fat and high-cholesterol (60% energy from fat and 1.25% cholesterol) diet containing sodium cholate induced NASH in mice, and the pathological features of this model were similar to those of humans, such as steatohepatitis, inflammation, fibrosis [17], lipid metabolism, insulin resistance, and hepatic gene expression profiles responsible for liver pathology in a mouse model [18]. These findings showed that a high-fat, high-cholesterol, and cholate-containing diet induced a pathology that resembled human NASH more than that induced by the MCD diet. However, this diet also results in severe body weight loss, and the main lipid in the liver is cholesterol, not triglycerides (TG). Another high-fat diet containing *trans* fatty acid and cholesterol induced obesity, insulin resistance, and NASH, but took 30 weeks to establish NASH in mice [19]. A high-fat (40% energy) diet containing *trans* fatty acids (36.3% of total fat) for 16 weeks induced insulin resistance and NASH in low density lipoprotein receptor knockout mice; however, these mice had less adiposity and weight gain than those fed a diet rich in polyunsaturated fatty acids or saturated fatty acids [20]. The Joint WHO/FAO Expert Consultation on Diet, Nutrition, and the Prevention of Chronic Diseases has recommended that the intake of *trans* fats be reduced (less than 1% of energy intake). Therefore, a *trans* fat-containing diet is not ideal for producing a dietary model of NASH.

As each diet has advantages and disadvantages, we attempted to identify a diet that induces the pathology of NASH without weight loss. In the present study, C57BL/6 mice were fed a high-fat and choline-deficient (HFCD) diet containing several doses of methionine for 12 weeks, and body weight gain and progression of NASH in mice were evaluated. More than 0.2% methionine in the HFCD diet was necessary for body weight gain, and less than 0.2% methionine in the HFCD diet was able to induce NASH after 12 weeks in mice.

## Materials and Methods

### Animals and experimental design

Male C57BL/6J mice (9 weeks of age) were purchased from CLEA Japan Inc. (Tokyo, Japan). After 1 week of acclimation, mice (10 weeks of age) were fed an L-amino acid rodent (control) diet, high-fat (HF) diet, or HFCD diet containing various amounts of methionine (0.1–0.6% (w/w)). A level of 0.6% methionine is the same as in the control and HF diets. The exact compositions of each diet are shown in S1 Table. These diets were purchased from Research Diet Inc. (New Brunswick, NJ). Mice were housed in cages (less than 5 mice in each cage) with a 12-hour light/dark cycle, and were given food and water *ad libitum*. All animal experiments were conducted with the approval of the National Institutes of Biomedical Innovation, Health and Nutrition Laboratory Animal Ethics Committee (No. 1413).

### Plasma chemistry

After 8 or 12 weeks of feeding, mice were fasted overnight and killed under isoflurane anesthesia. Blood samples were obtained from the heart, and plasma samples were prepared immediately. Plasma levels of total cholesterol (TC), TG, non-esterified fatty acids (NEFA), glucose and liver functional markers (GOT, GPT and alkaline phosphatase (ALP)) were measured using enzymatic methods (Wako Pure Chemical Industries, Osaka, Japan). Plasma levels of insulin were measured using the Mercodia Ultrasensitive Mouse Insulin ELISA kit (Mercodia, Uppsala, Sweden).

### Measurement of hepatic lipid content

Hepatic lipids were extracted with chloroform/methanol (2:1) using Folch’s method [21]. The liver (approx. 100 mg) was homogenized in 6 mL of chloroform/methanol (2:1) with a polytron homogenizer. Lipids were extracted by overnight incubation, filtrated to remove insoluble residues, and adjusted to 10 mL with chloroform/methanol. One milliliter of each sample was added to a microtube and the organic solvent was evaporated. The residue was dissolved in isopropanol (with 10% Triton-X). TC, TG and NEFA contents were measured using enzymatic methods (Wako Pure Chemical Industries).

### Histological examination

Livers were removed and part of each liver was immediately fixed with 10% buffered formalin solution. Liver samples were embedded in paraffin and sectioned at a thickness of 3 μm for hematoxylin and eosin (HE) and Sirius red staining. NAFLD activity scores were determined by steatosis, lobular inflammation and hepatocyte ballooning scores. The area of fibrosis was also measured. The pathological features of steatosis (0–3), lobular inflammation (0–3), hepatocyte ballooning (0–2) and fibrosis (0–4) were scored by Allisere, Inc. (Tokyo, Japan). Mice with an NAFLD activity score of more than 5 were diagnosed as “NASH”, between 5 to 3 as “borderline”, and less than 3 as “not NASH” [22]. F4/80 immuno-staining was performed with a rat anti-mouse F4/80 antibody (A3-1; BIO-RAD Laboratories, Inc., Hercules, CA) as a primary antibody, rabbit polyclonal rat IgG-heavy and light chain cross-absorbed antibody (A110-322A; BETHYL Laboratories, Inc., Montgomery, TX) as a secondary antibody, and a BOND Polymer Refine Detection System (LEICA Biosystems, Nussloch, Germany).

### Quantitative RT-PCR

Total RNA was extracted from the liver using the TRIzol Plus RNA Purification System (Life Technologies, Carlsbad, CA), and reverse transcribed with the PrimeScript RT Master Mix (Takara Bio Inc., Shiga, Japan). Quantitative RT-PCR was performed in 96-well plates with the SYBR Green PCR Master Mix and a Thermal Cycler Dice Real Time System Single (Takara Bio Inc.). Copy numbers for each gene were determined by the absolute quantitation method using serially diluted standards of known concentrations. Results are expressed as the copy number ratio of the target mRNA to GAPDH mRNA. The mouse-specific primer pairs used in this experiment are shown in S2 Table.

### Intraperitoneal glucose tolerance test

After overnight fasting, D-glucose solution (2 g/kg BW) was administered by intraperitoneal injection. Controls were given saline. Blood glucose levels were monitored by tail bleeding prior to administration of glucose (0 minute), and at 15, 30, 60, 120 and 180 minutes after glucose injection using Breeze2 (Bayer Pharma AG, Berlin, Germany).

### Statistical analyses

Data are presented as means ± SEM. Comparisons of data from multiple groups were performed by one-way ANOVA with a Bonferroni post-hoc test (SPSS 18.0J for Windows; IBM Co., Armonk, NY). Comparisons of data between two groups were performed by unpaired Student’s *t*-test (PASW Statistics Base 18; IBM). A *P* value < 0.05 was considered to be significant.

## Results

### Preliminary study

C57BL/6J mice (male, 10 weeks of age) were fed a control diet, HF diet or HFCD diet containing 0.1% (LM) or 0.6% methionine (HM) for 4, 8 and 12 weeks. The HFCD diet without methionine has already been shown to induce body weight loss in mice. Therefore, we did not use the HFCD diet without methionine in our experiments. The body weight of mice fed the LM-HFCD diet was lower than that of mice fed the control diet. On the other hand, the body weight of mice fed the HM-HFCD diet was similar to that of mice fed the HF diet (S1A Fig). Under these conditions, liver weight, plasma levels of GOT and GPT, and hepatic TC and TG levels were significantly higher in mice fed the LM-HFCD diet than in those fed the control or HF diet. On the other hand, mice fed the HM-HFCD diet showed almost the same changes in these parameters as mice fed the HF diet (S1B–S1G Fig). These results suggest that a choline deficiency is not sufficient to induce the progression of NASH, and that 0.1% methionine is not adequate to attenuate body weight loss in mice fed the HFCD diet. At 0.1% methionine, these parameters reached a maximum at 8 weeks and slightly decreased at 12 weeks. Therefore, we investigated the dose-dependent effects of methionine on body weight gain and NASH progression for 8 weeks or 12 weeks.

### 8-week feeding

#### HFCD diet containing more than 0.2% methionine increased body weight, but did not induce NASH

C57BL/6J mice (male, 10 weeks of age) were fed the control diet, HF diet or HFCD diet containing 0.1, 0.2, 0.4 and 0.6% methionine for 8 weeks. Body weight gain was lower in mice fed the 0.1% methionine in HCFD diet than that those fed the control diet, and increased in a methionine dose-dependent manner (Table 1). Body weight gain in mice fed the 0.4% and 0.6% methionine in HFCD diets was similar to that in mice fed the HF diet. On the other hand, liver weight and the % liver weight, and liver injury (GOT, GPT and ALP levels) were greatest in mice fed the 0.1% methionine in HFCD diet, and decreased in a methionine dose-dependent manner (Table 1). Under these conditions, hepatic TC and TG contents (S2 Fig), gene expression levels (S3 Fig), and pathological analysis (S4 Fig) showed that lower methionine content in the HFCD diet induced the pathology of NASH. However, NAFLD activity score indicated that only mice fed the 0.1% methionine in HFCD diet developed NASH (NAFLD activity score was more than 5), those fed the 0.2% and 0.4% methionine in HFCD diets were borderline (from 3 to 5), and those fed the 0.6% methionine in HFCD diet did not develop NASH (less than 3) (Table 2).

**Table 1:** Body Weight, Tissue Weights and Plasma Biomarkers (8 Weeks).

	Control	HF	HFCD + 0.1% Met	HFCD + 0.2% Met	HFCD + 0.4% Met	HFCD + 0.6% Met
Body weight (g)	25.8 ± 0.5	33.1 ± 1.1***	22.1 ± 0.6	27.6 ± 0.9	33.4 ± 1.1***	31.5 ± 1.2**
Liver (g)	0.99 ± 0.02	1.07 ± 0.04	1.54 ± 0.11***	1.47 ± 0.05***	1.21 ± 0.05	1.08 ± 0.03
Liver/BW (%)	3.84 ± 0.07	3.25 ± 0.09	6.90 ± 0.31***	5.32 ± 0.12***	3.60 ± 0.06	3.46 ± 0.11
Kidney (g)	0.31 ± 0.01	0.37 ± 0.01***	0.28 ± 0.01	0.33 ± 0.01	0.35 ± 0.01*	0.34 ± 0.01
Spleen (g)	0.060 ± 0.003	0.074 ± 0.005	0.073 ± 0.005	0.073 ± 0.003	0.072 ± 0.004	0.065 ± 0.004
Visceral Fat (g)	0.59 ± 0.05	1.65 ± 0.15***	0.35 ± 0.04	0.85 ± 0.11	1.74 ± 0.13***	1.45 ± 0.17***
Plasma biomarkers						
TC (mg/dL)	121.7 ± 3.8	135.9 ± 6.8	63.1 ± 3.0***	107.1 ± 3.7	134.3 ± 4.2	129.6 ± 4.1
TG (mg/dL)	157.5 ± 17.2	130.5 ± 11.0	89.2 ± 4.0**	121.5 ± 1.5	132.2 ± 8.8	97.2 ± 7.9**
NEFA (mEq/L)	1.92 ± 0.26	1.39 ± 0.12	1.08 ± 0.10**	1.37 ± 0.09	1.28 ± 0.05	1.15 ± 0.05**
Glucose (mg/dL)	89.1 ± 4.6	134.3 ± 7.9**	110.8 ± 6.0	107.9 ± 7.2	121.6 ± 13.0	120.9 ± 8.7
Insulin (ng/L)	30.0 ± 4.1	39.2 ± 4.5	22.2 ± 1.0	24.9 ± 2.0	34.6 ± 3.8	28.4 ± 2.2
GOT (IU/L)	49.1 ± 3.5	47.4 ± 3.2	109.0 ± 8.4***	78.5 ± 1.8**	59.6 ± 6.5	57.1 ± 4.7
GPT (IU/L)	6.7 ± 1.4	7.1 ± 1.1	58.1 ± 8.3***	43.4 ± 5.8***	18.0 ± 2.9	10.1 ± 2.0
ALP (IU/L)	48.1 ± 1.1	39.0 ± 2.0*	69.7 ± 3.5***	54.8 ± 2.0	38.7 ± 1.5*	38.6 ± 1.6*

C57BL/6J mice were fed each experimental diet for 8 weeks. After overnight fasting, body weight and tissue weights were measured. Blood samples were withdrawn using heart puncture, and plasma samples were collected. Plasma levels of TC, TG, NEFA, glucose, insulin, GOT, GPT, and ALP were measured. HF; high-fat diet, HFCD; high-fat, choline-deficient diet; Met; methionine. Data are represented as the mean ± SEM. n = 7 or 8 in each diet.

*P <0.05

**P <0.01

***P <0.001 vs. control.

**Table 2:** NAFLD Activity Score (8 Weeks).

	Control	HF	HFCD + 0.1% Met	HFCD + 0.2% Met	HFCD + 0.4% Met	HFCD + 0.6% Met
Steatosis	0.00 ± 0.00	0.71 ± 0.18	3.00 ± 0.00***	2.29 ± 0.36***	1.71 ± 0.29***	1.14 ± 0.34**
Inflammation	0.14 ± 0.14	0.86 ± 0.34	2.43 ± 0.20***	2.00 ± 0.31***	1.43 ± 0.37*	1.43 ± 0.30*
Ballooning	0.00 ± 0.00	0.14 ± 0.14	0.00 ± 0.00	0.00 ± 0.00	0.14 ± 0.14	0.14 ± 0.14
NAFLD Activity Score	0.14 ± 0.14	1.71 ± 0.52	5.43 ± 0.20***	4.29 ± 0.52***	3.29 ± 0.64***	2.71 ± 0.57**

C57BL/6J mice were fed each experimental diet for 8 weeks. After overnight fasting, mice were killed and the liver was removed. The liver was fixed with 10% buffered formalin solution, embedded in paraffin, cut into 4-μm-thick sections, and stained with hematoxylin-eosin or Sirius red. HF; high-fat diet, HFCD; high-fat, choline-deficient diet; Met; methionine. Data are represented as the mean ± SEM. n = 7 in each diet.

*P <0.05

**P <0.01

***P <0.001 vs. control.

### 12-week feeding

#### HFCD-suppressed body weight gain and -induced liver damage was attenuated in a methionine dose-dependent manner

**Fig 1:**
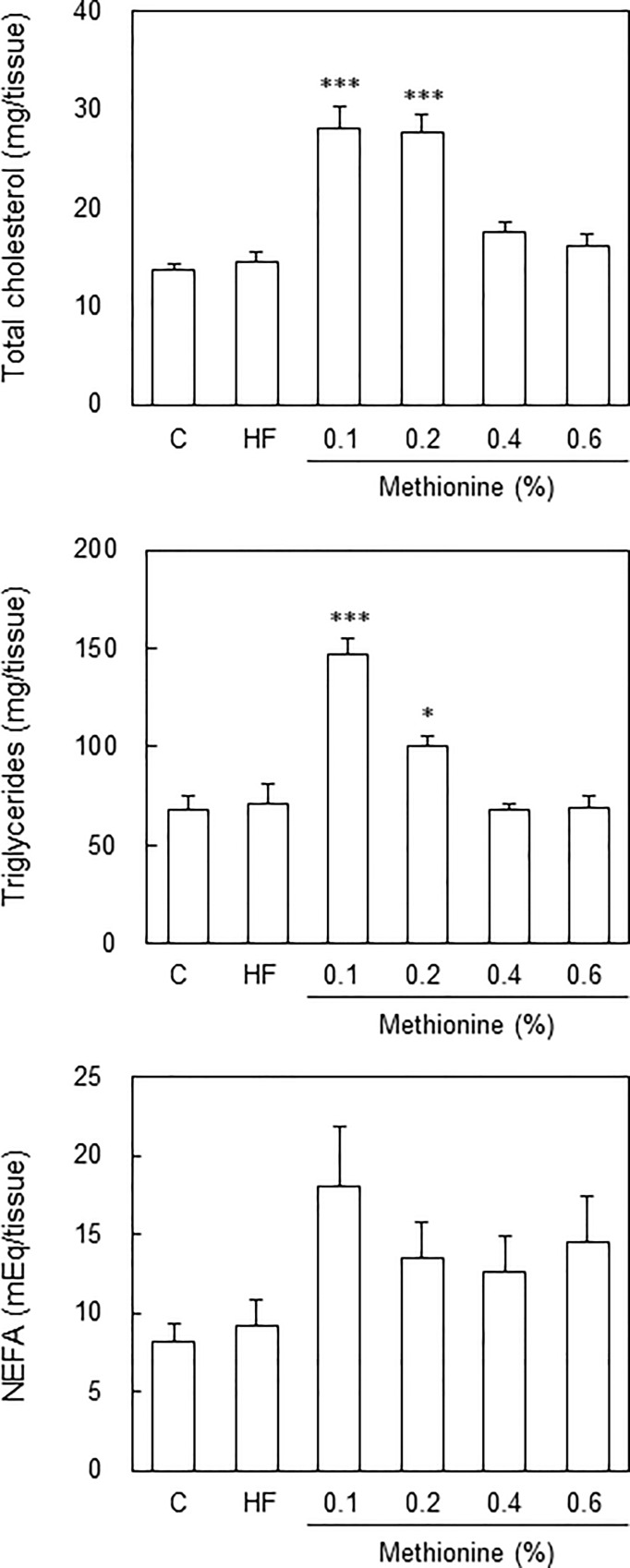
Hepatic lipid contents in each diet. C57BL/6J mice (male, 10 weeks of age) were fed a control diet, HF diet, or HFCD diet containing 0.1%, 0.2%, 0.4% or 0.6% methionine for 12 weeks. After overnight fasting, mice were killed and the liver was removed. Hepatic lipids were extracted according to Folch’s method, and TC, TG and NEFA levels were measured using enzymatic methods. *P < 0.05, ***P < 0.001 vs. control by a one-way ANOVA with Bonferroni’s post-hoc test.

When fed each diet for 8 weeks, only mice fed the 0.1% methionine in HFCD diet were diagnosed with NASH; however, this diet suppressed body weight gain. On the other hand, mice fed the 0.2% and 0.4% methionine in HFCD diets were diagnosed as borderline, and gained body weight. Therefore, we extended the feeding period to 12 weeks. When fed each diet for 12 weeks, body weight in mice fed the 0.1% methionine in HFCD diet was still lower than that in mice fed the control, and it increased in a methionine dose-dependent manner (Table 3). Similar to the results obtained with 8 weeks of feeding, body weight in mice fed the 0.2% methionine in HFCD diet was similar to that in mice fed the control diet, while body weight in mice fed the 0.4% and 0.6% methionine in HFCD diets was similar to that in mice fed the HF diet. Liver weight and % liver weight, and liver injury (GOT, GPT, and ALP levels) were greatest in mice fed the 0.1% methionine in HFCD diet, and these increased in a methionine dose-dependent manner. Plasma levels of TC, but not TG were significantly lower in mice fed the 0.1% methionine in HFCD diet than in those fed the control diet (Table 3). Hepatic TC and TG levels were significantly higher in mice fed the 0.1% and 0.2% methionine in HFCD diets (Fig 1). No significant differences were observed in plasma glucose or insulin levels between mice fed the 0.1% and 0.2% methionine in HFCD diets and those fed the control diet; however, these levels increased in a methionine dose-dependent manner. These results suggest that the HFCD diet with methionine induces insulin resistance, similar to the HF diet, whereas decreasing the methionine content in the HFCD diet suppresses it.

**Table 3:** Body Weight, Tissue Weights and Plasma Biomarkers (12 Weeks).

	Control	HF	HFCD + 0.1% Met	HFCD + 0.2% Met	HFCD + 0.4% Met	HFCD + 0.6% Met
Body weight (g)	28.7 ± 1.1	37.9 ± 1.7***	23.3 ± 0.7	30.1 ± 1.2	35.7 ± 1.6*	36.5 ± 1.9**
Liver (g)	1.06 ± 0.04	1.14 ± 0.07	1.71 ± 0.10***	1.54 ± 0.06***	1.26 ± 0.05	1.21 ± 0.07
Liver/BW (%)	3.69 ± 0.07	3.01 ± 0.09**	7.33 ± 0.22***	5.11 ± 0.09***	3.58 ± 0.10	3.33 ± 0.08
Kidney (g)	0.33 ± 0.01	0.38 ± 0.01*	0.30 ± 0.01	0.34 ± 0.01	0.36 ± 0.01	0.36 ± 0.01
Spleen (g)	0.069 ± 0.003	0.080 ± 0.004	0.088 ± 0.006*	0.079 ± 0.002	0.077 ± 0.003	0.078 ± 0.003
Visceral Fat (g)	0.93 ± 0.12	2.28 ± 0.21***	0.50 ± 0.04	1.44 ± 0.16	2.18 ± 0.23***	2.19 ± 0.25***
Plasma biomarkers						
TC (mg/dL)	132.1 ± 7.0	145.7 ± 9.8	62.7 ± 3.6***	111.1 ± 5.6	127.9 ± 8.3	136.8 ± 7.7
TG (mg/dL)	138.4 ± 9.1	93.9 ± 5.9***	100.4 ± 4.7**	115.0 ± 5.7	96.7 ± 6.9**	84.1 ± 7.3***
NEFA (mEq/L)	2.07 ± 0.24	1.22 ± 0.05***	1.22 ± 0.09***	1.28 ± 0.04***	1.24 ± 0.09***	1.18 ± 0.10***
Glucose (mg/dL)	109.9 ± 12.2	186.6 ± 16.0***	114.4 ± 5.6	118.3 ± 6.5	148.5 ± 12.1	142.2 ± 15.5
Insulin (ng/L)	24.8 ± 1.5	201.0 ± 86.2	44.0 ± 12.5	29.9 ± 2.9	144.7 ± 87.5	178.5 ± 89.3
GOT (IU/L)	59.2 ± 9.4	59.8 ± 4.1	130.6 ± 8.9***	92.8 ± 3.9*	66.1 ± 2.5	63.2 ± 8.9
GPT (IU/L)	6.7 ± 1.0	13.3 ± 2.0	45.0 ± 4.5***	44.2 ± 6.4***	25.6 ± 4.3	17.3 ± 3.7
ALP (IU/L)	49.7 ± 2.0	41.6 ± 2.8	73.8 ± 2.0***	55.9 ± 1.8	40.8 ± 1.6	41.0 ± 1.8

C57BL/6J mice were fed each experimental diet for 12 weeks. After overnight fasting, body weight and tissue weights were measured. Blood samples were withdrawn using heart puncture, and plasma samples were collected. Plasma levels of TC, TG, NEFA, glucose, insulin, GOT, GPT, and ALP were measured. HF; high-fat diet, HFCD; high-fat, choline-deficient diet; Met; methionine. Data are represented as the mean ± SEM. n = 7 or 8 in each diet.

*P <0.05

**P <0.01

***P <0.001 vs. control.

#### HFCD-induced inflammatory and fibrosis marker gene expression was attenuated in a methionine dose-dependent manner

**Fig 2:**
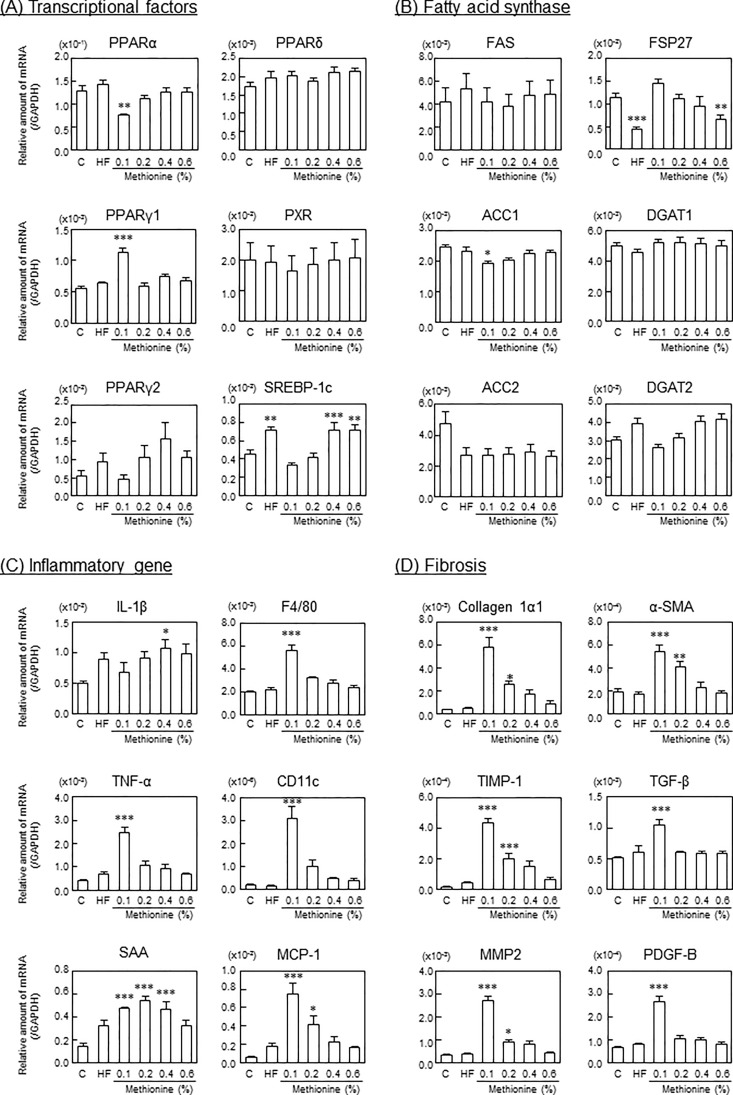
Gene expression levels in liver. C57BL/6J mice (male, 10 weeks of age) were fed each experimental diet for 12 weeks. Total RNA was extracted from the liver, and expression levels of (A) transcriptional factors, (B) fatty acid synthase related genes, (C) inflammatory related genes, and (D) fibrosis-related genes were measured by real-time RT-PCR methods. Data are represented as means and SEM, n = 7 or 8 in each diet. *P < 0.05, **P < 0.01, ***P < 0.001 vs. control by a one-way ANOVA with Bonferroni’s post-hoc test.

At 12 weeks, PPARα expression levels were significantly lower, while PPARγ1 mRNA expression levels were significantly higher in mice fed the 0.1% methionine in HFCD diet than in those fed the control diet. No significant differences were observed in PPARγ2 or SREBP-1c levels between mice fed the 0.1% methionine in HFCD diet and those fed the control diet; however, these levels increased in a methionine dose-dependent manner, similar to the HF diet (Fig 2A). Although FSP27 gene expression levels were not significantly different between mice fed the 0.1% methionine in HFCD diet and those fed the control diet, they decreased in a methionine dose-dependent manner, similar to the HF diet. On the other hand, DGAT2 levels increased in a methionine dose-dependent manner, similar to the HF diet (Fig 2B). Under these conditions, inflammatory cytokine (TNF-α), inflammatory marker protein (serum amyloid A (SAA)), macrophage markers (F4/80 and CD11c) and monocyte chemotactic protein (MCP)-1 mRNA expression levels were greater in mice fed the 0.1% and 0.2% methionine in HFCD diets than in those fed the control and HF diets, and these changes were attenuated by increasing the methionine content. Increased CD11c mRNA levels indicated that macrophages in mice fed the 0.1% and 0.2% methionine in HFCD diet were M1 macrophages (Fig 2C). Furthermore, fibrosis marker (collagen type 1α1, tissue inhibitor of metalloproteinase (TIMP)-1, matrix metalloproteinase (MMP)-2, α-smooth muscle actin (SMA), transforming growth factor (TGF)-β and platelet-derived growth factor (PDGF)-B) mRNA expression levels were also greater in mice fed the 0.1% and 0.2% methionine in HFCD diets than in those fed the control and HF diets (Fig 2D).

#### Pathological analysis and NAFLD activity scores

**Fig 3:**
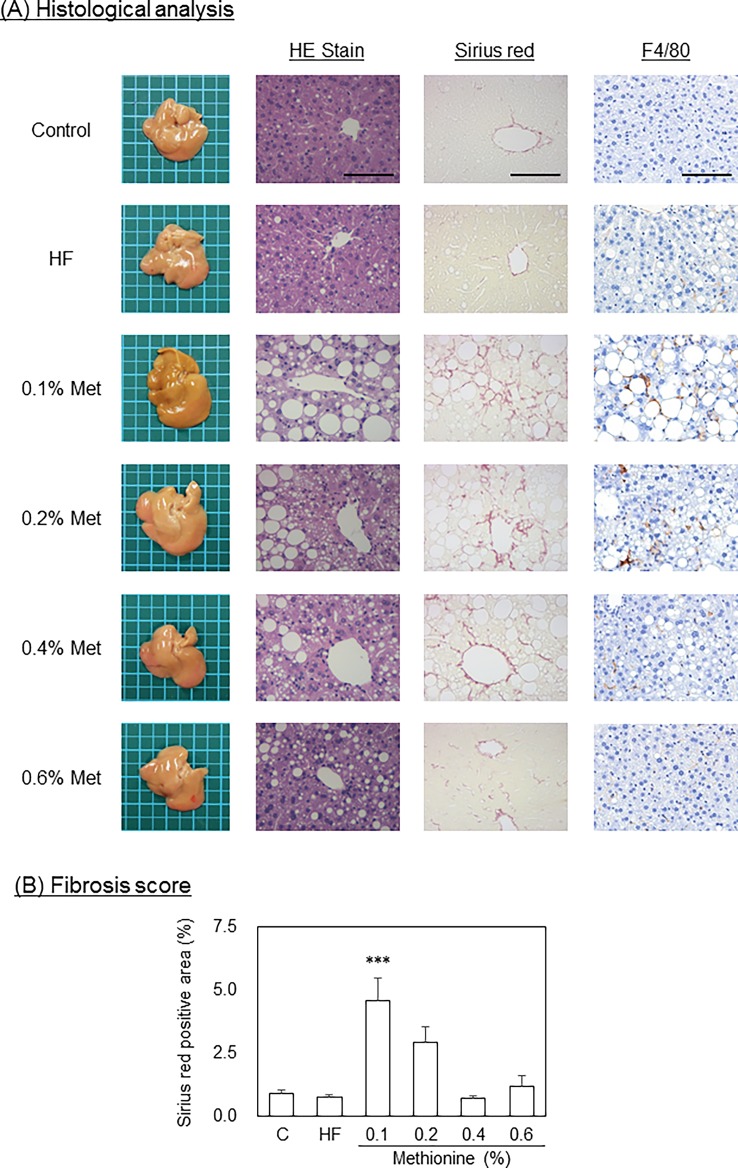
Pathological analysis. C57BL/6J mice (male, 10 weeks of age) were fed each experimental diet for 12 weeks. After overnight fasting, mice were killed and the liver was removed. (A) A pathological analysis with HE staining, serial red staining and F4/80 immuno-staining was conducted. Bar indicates 100 μm. Original magnification x200. (B) Fibrosis score was determined as the ratio of sirius red-positive area to the whole area in each section. Data are represented as means and SEM, n = 7 or 8 in each diet. ***P < 0.01 vs. control by one-way ANOVA with Bonferroni’s post-hoc test.

Macrovesicular steatosis was seen in mice fed the 0.1%, 0.2% and 0.4% methionine in HFCD diet. On the other hand, microvesicular steatosis was detected in mice fed the HF diet (Fig 3). Steatosis scores were significantly greater in mice fed the HF diet and HFCD diets than in those fed the control diet. In addition, among the HFCD diets tested, the steatosis score was the highest in mice fed the 0.1% methionine in HFCD diet, and decreased in a methionine dose-dependent manner (Table 4). The inflammation score was also significantly greater in mice fed the HF diet and the 0.1% and 0.2% methionine in HFCD diets (Fig 2 and Table 4). The severity of fibrosis was significantly greater in mice fed the 0.1% and 0.2% methionine in HFCD diets than in those fed the control diet, and that in mice fed the 0.1% methionine in HFCD diet was the highest among the HFCD diets examined (Fig 3A and 3B). However, the ballooning score remained low in all groups. Based on NAFLD activity at 12 weeks, mice fed the 0.1% and 0.2% methionine in HFCD diets were diagnosed as NASH, those fed the 0.4% methionine in HFCD diet were borderline, and those fed the 0.6% methionine in HFCD diet did not have NASH.

**Table 4:** NAFLD Activity Score (12 weeks).

	Control	HF	HFCD + 0.1% Met	HFCD + 0.2% Met	HFCD + 0.4% Met	HFCD + 0.6% Met
Steatosis	0.14 ± 0.14	2.14 ± 0.26***	3.00 ± 0.00***	2.75 ± 0.16***	2.17 ± 0.31***	1.57 ± 0.20***
Inflammation	0.29 ± 0.18	1.86 ± 0.40**	2.86 ± 0.14***	2.38 ± 0.26***	0.83 ± 0.17	1.29 ± 0.36
Ballooning	0.14 ± 0.14	0.14 ± 0.14	0.14 ± 0.14	0.00 ± 0.00	0.00 ± 0.00	0.00 ± 0.00
NAFLD Activity Score	0.57 ± 0.20	4.14 ± 0.63***	6.00 ± 0.22***	5.13 ± 0.30***	3.00 ± 0.26***	2.86 ± 0.34***

C57BL/6J mice were fed each experimental diet for 12 weeks. After overnight fasting, mice were killed and the liver was removed. The liver was fixed with 10% buffered formalin solution, embedded in paraffin, cut into 4-μm-thick sections, and stained with hematoxylin-eosin or Sirius red. HF; high-fat diet, HFCD; high-fat, choline-deficient diet; Met; methionine. Data are represented as the mean ± SEM. n = 7 in each diet.

*P <0.05

**P <0.01

***P <0.001 vs. control.

#### The 0.2% methionine in HFCD diet did not induce glucose intolerance

**Fig 4:**
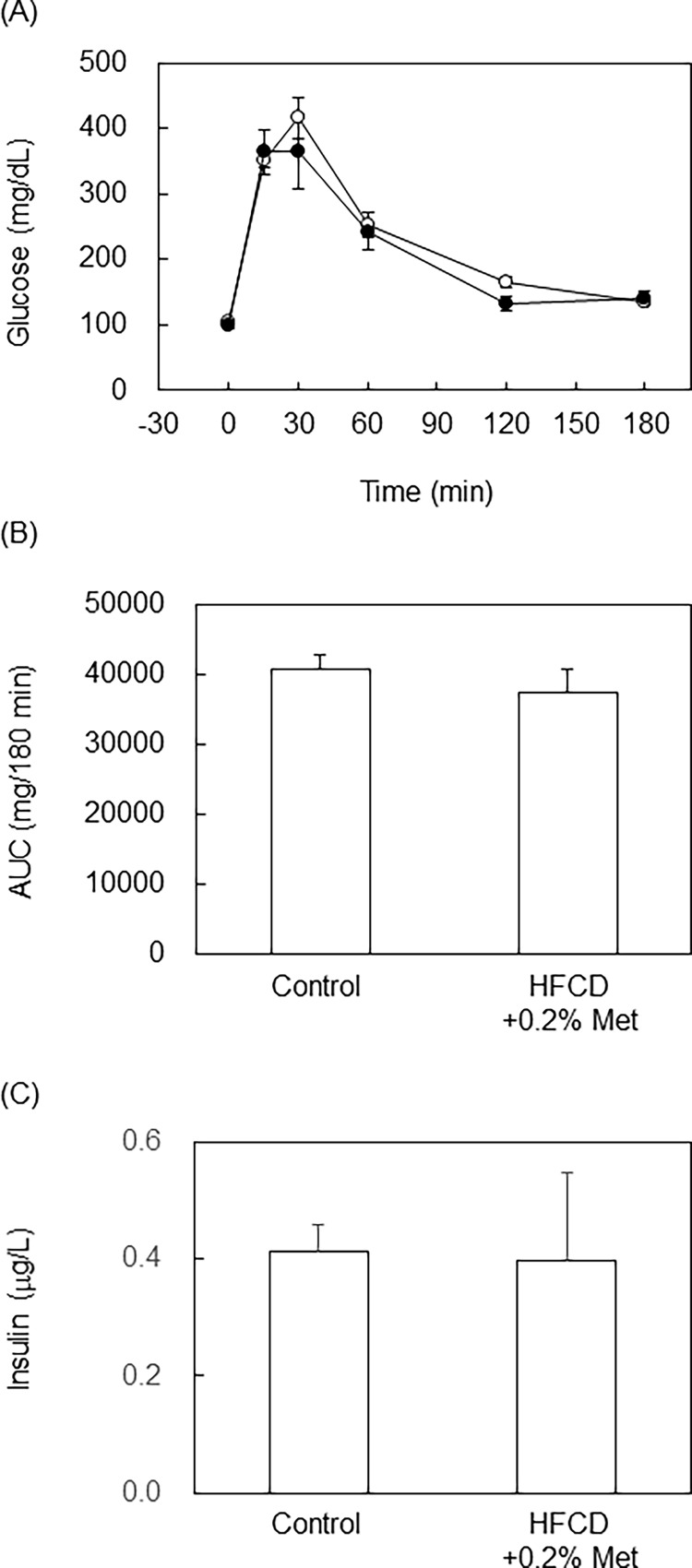
IPGTT was conducted in mice fed 0.2% methionine in HFCD diet for 12 weeks. C57BL/6J mice (male, 10 weeks of age) were fed a control diet or 0.2% methionine in HFCD diet for 12 weeks, and IPGTT was conducted after overnight fasting. (A) After injection of glucose (2 g/kg BW, i.p.), plasma glucose levels were measured at each time point. (B) AUC was calculated in (A). (C) Plasma insulin levels were measured using an ELISA kit. n = 6 in each diet.

Mice fed the 0.2% methionine in HFCD diet for 12 weeks developed the pathology of NASH without body weight gain being suppressed. Therefore, we conducted IPGTT on mice fed the 0.2% methionine in HFCD diet for 12 weeks. However, no significant differences were observed in IPGTT, AUC or plasma insulin levels between mice fed the control diet and those fed the 0.2% methionine in HFCD diet (Fig 4). These results suggest that the 0.2% methionine in HFCD diet did not induce glucose intolerance, similar to the MCD diet, even though body weight gains were observed.

## Discussion

The most commonly used diet to induce NASH in rodents is the MCD diet; however, it causes severe weight loss, which differs from human NASH. A previous study demonstrated that a methionine and choline deficiency induced weight loss even with a HF diet [15]. In order to overcome this issue, we examined the effects of methionine supplementation with a HFCD diet on body weight and NASH pathology. In the present study, body weight in mice fed the 0.1% methionine in HFCD diet was lower than that in mice fed control diet, whereas more than 0.2% methionine induced body weight gain. More than 0.4% methionine for 12 weeks may induce obesity, but not the pathology of NASH. Therefore, the 0.2% methionine in HFCD diet for 12 weeks is suitable for inducing NASH without decreasing body weight in mice.

Choline is important as a source of phosphatidylcholine (PC). PC is an essential component of very low density lipoprotein, which plays an important role in the secretion of TG from the liver [23]. Therefore, the impaired synthesis of PC due to choline deficiency causes the accumulation of lipids in the liver. Methionine is another source of PC. A sufficient amount of methionine (more than 0.4%) in the HFCD diet did not significantly alter lipid accumulation from that observed with the HF diet in this experiment. Furthermore, methionine is an essential amino acid and plays an important role in protein synthesis and liver function [24]; thus, methionine deficiency induces NASH as a second hit in a choline-deficient diet [25]. Furthermore, methionine has been linked to body weight gain. Body weight gain was shown to be significantly lower with methionine restriction than with a control diet in mice [26] and rats [27]. A previous study directly compared control, methionine-deficient (MD), choline-deficient (CD), and MCD diets [28]. During 15 days of feeding, the CD diet induced body weight gain and did not affect serum ALT levels. On the other hand, the MD and MCD diets reduced body weight and induced liver injury. Hepatic TG accumulation was observed with the CD, MD and MCD diets, but was less prominent with the MD diet than with the CD and MCD diets. These findings and our results clearly demonstrate that methionine and choline play roles in body weight gain and liver injury.

PPARα is an important transcription factor in NASH. PPARα mRNA expression levels were significantly lower in mice fed the 0.1% methionine in HFCD diet and increased in a methionine dose-dependent manner. The MCD diet induced hepatic lipid accumulation by not only preventing the synthesis and secretion of VLDL from the liver, but by also preventing the β-oxidation of FFA in the liver. PPARα also has an anti-inflammatory property. A previous study reported that activation of NFκB and PPARα is reciprocally regulated by protein-protein interactions [29]; therefore, inflammation induced by the MCD diet may suppress PPARα mRNA expression via the activation of NFκB. Wy14,643, a PPARα gene agonist, improved steatosis and ballooning in diabetic NASH model mice [30], and a dual PPARα/δ agonist exerted liver-protective effects on steatosis, inflammation, and fibrosis in several rodent models [31]. In humans, liver PPARα gene expression negatively correlates with NASH severity [32]. Therefore, PPARα is attracting interest as a therapeutic target for NAFLD/NASH [33, 34].

HF diets containing *trans* fatty acids are also used to induce NASH in mouse models [19, 20]. In addition, cholesterol and cholate induced not only lipotoxicity in the liver, but also weight loss and reductions in adipose tissue, even though this diet contained 60% fat [18]. However, one of our aims is to establish a NASH-inducing diet that is as similar as possible to the human diet. Therefore, we did not add *trans* fatty acids, cholesterol, or sodium cholate to the diets examined. A HF diet, which induces obesity, typically contains 45–60% energy from fat, but 60% energy from fat in the diet is atypical for humans, even in Western countries, and we considered 46% energy from fat in our diet to be sufficient to induce obesity. On the other hand, the MCD diet only contains 20% energy from fat, and this diet decreased body weight and adipose tissue mass. In order to establish a NASH model without weight loss, we need to combine the features of the HF and MCD diets.

There are still some differences between the NASH induced by the 0.2% methionine in HFCD diet and human NASH. The 0.2% methionine in HFCD diet was found to only weakly induce ballooning in the pathological analysis of the liver. Furthermore, IPGTT and plasma insulin levels showed that mice fed the 0.2% methionine in HFCD diet for 12 weeks did not develop insulin resistance. These phenotypes have been observed in mice fed the MCD diet [12, 35]. Under our experimental conditions, mice fed the 0.2% methionine in HFCD diet for 12 weeks gained body weight similar to those fed the control diet; however, they were not obese. On the other hand, the 0.4% methionine in HFCD diet induced obesity, but not NASH. However, some of the phenotypes were intermediate between the 0.2% and 0.6% methionine in HFCD diets. Therefore, increasing the methionine content to 0.3–0.4% in the HFCD diet and extending feeding periods may be needed in order to induce obesity and insulin resistance with NASH. Another possibility to induce insulin resistance with NASH is fructose supplementation. A previous study reported that feeding C57BL/6 mice a high-fat (40% energy) diet containing 2% cholesterol with fructose for 25 weeks induced insulin resistance and steatohepatitis [36]. Therefore, supplementation of fructose in drinking water with the 0.2% methionine in HFCD diet may induce obesity and NASH within 12 weeks.

## Conclusions

The results of the present study indicated that at least 0.2% methionine is needed in the HFCD diet in order for mice to gain body weight. On the other hand, HFCD diet-induced hepatic damage was attenuated in a methionine dose-dependent manner, and less than 0.2% methionine induced the pathology of NASH in mice after 12 weeks. The 0.2% methionine in HFCD diet for 12 weeks was able to induce NASH without weight loss.

## Supporting Information

S1 Fig Preliminary study of influence of methionine in HFCD diet on body weight and progression of NASH. C57BL/6J mice (male, 10 weeks of age) were fed a control diet, HF diet, or HFCD diet containing 0.1% methionine (LM) or 0.6% methionine (HM) for 4, 8 and 12 weeks. (A) Body weight was measured every week. Open circles, control; closed circles, HF; open triangles, LM+HFCD; closed triangles, HM+HFCD. After each feeding period, mice were killed and (B) liver weight, (C) epididymal adipose tissue weight, (D) GOT, (E) GPT, (F) hepatic TC and (G) hepatic TG levels were measured. Open column, control; light gray column, HF; dark gray column, LM+HFCD; closed column, HM+HFCD. Data are represented as means and SEM, n = 5 in each diet. *P < 0.05, **P < 0.01, ***P < 0.001 vs. control.
(TIF)

S2 Fig Hepatic lipid contents in each diet (8 week feeding). C57BL/6J mice (male, 10 weeks of age) were fed a control diet, HF diet or HFCD diet containing 0.1%, 0.2%, 0.4% or 0.6% methionine for 8 weeks. After overnight fasting, mice were killed and the liver was removed. Hepatic lipids were extracted according to Folch’s method, and TC, TG and NEFA levels were measured by enzymatic methods. Data are represented as means and SEM, n = 7 or 8 in each diet. ***P < 0.001 vs. control by one-way ANOVA with Bonferroni’s post-hoc test.
(TIF)

S3 Fig Gene expression levels in liver (8 week feeding). C57BL/6J mice (male, 10 weeks of age) were fed each experimental diet for 8 weeks. Total RNA was extracted from the liver, and expression levels of (A) transcriptional factors, (B) fatty acid synthase-related genes, (C) inflammatory-related genes and (D) fibrosis-related genes were measured by real-time RT-PCR methods. Data are represented as mean and SEM, n = 7 or 8 in each diet. *P < 0.05, **P < 0.01, ***P < 0.001 vs. control by one-way ANOVA with Bonferroni’s post-hoc test.
(TIF)

S4 Fig Pathological analysis (8 week feeding). C57BL/6J mice (male, 10 weeks of age) were fed each experimental diet for 8 weeks. After overnight fasting, mice were killed and the liver was removed. (A) A pathological analysis with HE staining, serial red staining, and F4/80 immuno-staining was conducted. Bar indicates 100 μm. Original magnification, x200. (B) Fibrosis score was determined as the ratio of sirius red positive area to the whole area in each section. Data are represented as means and SEM, n = 7 or 8 in each diet. *P < 0.05 vs. control by one-way ANOVA with Bonferroni’s post-hoc test.
(TIF)

S1 Table Composition of Experimental Diets Used in This Study. (DOCX)

S2 Table Primer Sequences Used in This Study. (DOCX)
