# Supplementary figures and images for: Evaluation of Methionine Content in a High-Fat and Choline-Deficient Diet on Body Weight Gain and the Development of Non-Alcoholic Steatohepatitis in Mice
Source: PLoS One. 2016 Oct 10;11(10):e0164191. doi: 10.1371/journal.pone.0164191 (PMC5056759; doi:10.1371/journal.pone.0164191)

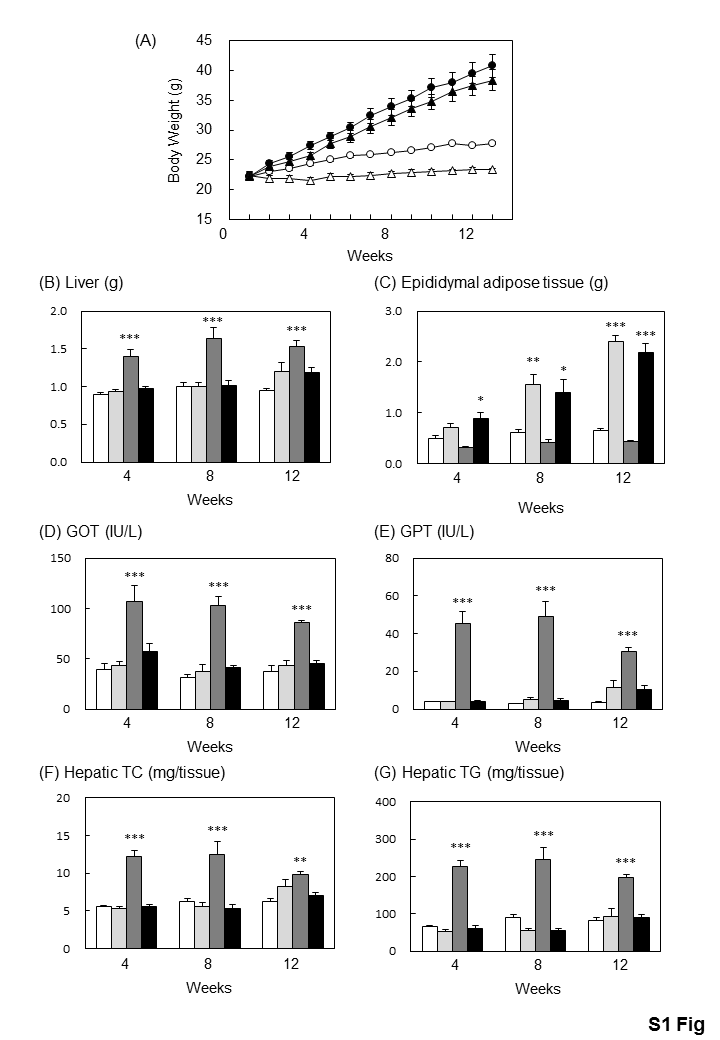

Supplement: S1 Fig — C57BL/6J mice (male, 10 weeks of age) were fed a control diet, HF diet, or HFCD diet containing 0.1% methionine (LM) or 0.6% methionine (HM) for 4, 8 and 12 weeks. (A) Body weight was measured every week. Open circles, control; closed circles, HF; open triangles, LM+HFCD; closed triangles, HM+HFCD. After each feeding period, mice were killed and (B) liver weight, (C) epididymal adipose tissue weight, (D) GOT, (E) GPT, (F) hepatic TC and (G) hepatic TG levels were measured. Open column, control; light gray column, HF; dark gray column, LM+HFCD; closed column, HM+HFCD. Data are represented as means and SEM, n = 5 in each diet. *P < 0.05, **P < 0.01, ***P < 0.001 vs. control. (TIF) [file pone.0164191.s001.tif]

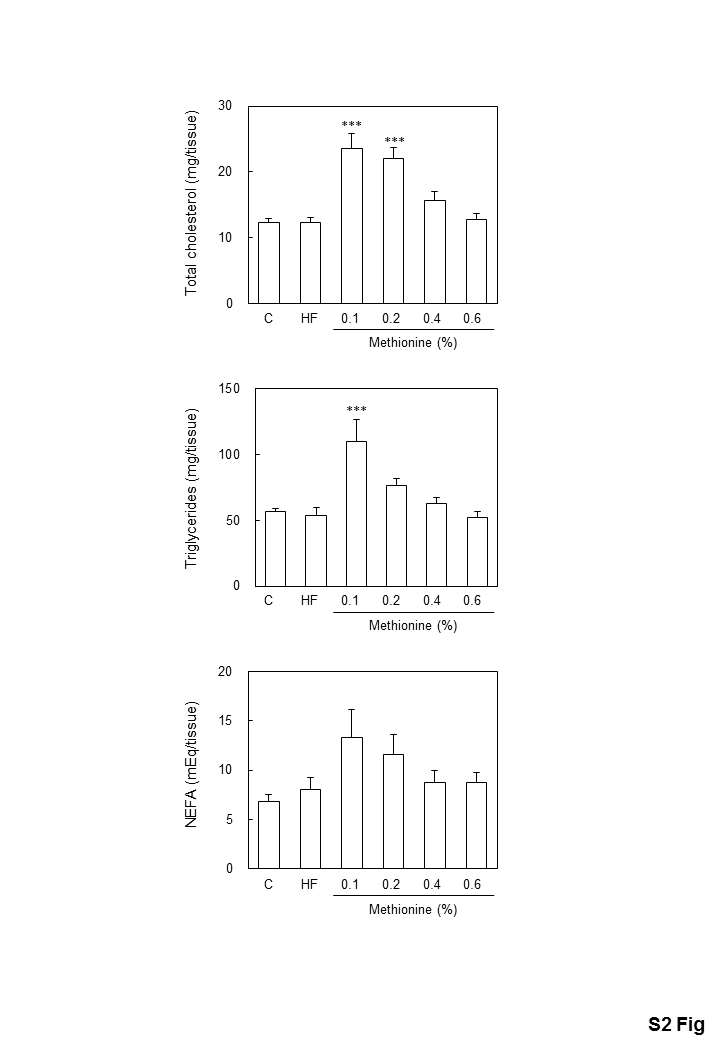

Supplement: S2 Fig — C57BL/6J mice (male, 10 weeks of age) were fed a control diet, HF diet or HFCD diet containing 0.1%, 0.2%, 0.4% or 0.6% methionine for 8 weeks. After overnight fasting, mice were killed and the liver was removed. Hepatic lipids were extracted according to Folch’s method, and TC, TG and NEFA levels were measured by enzymatic methods. Data are represented as means and SEM, n = 7 or 8 in each diet. ***P < 0.001 vs. control by one-way ANOVA with Bonferroni’s post-hoc test. (TIF) [file pone.0164191.s002.tif]

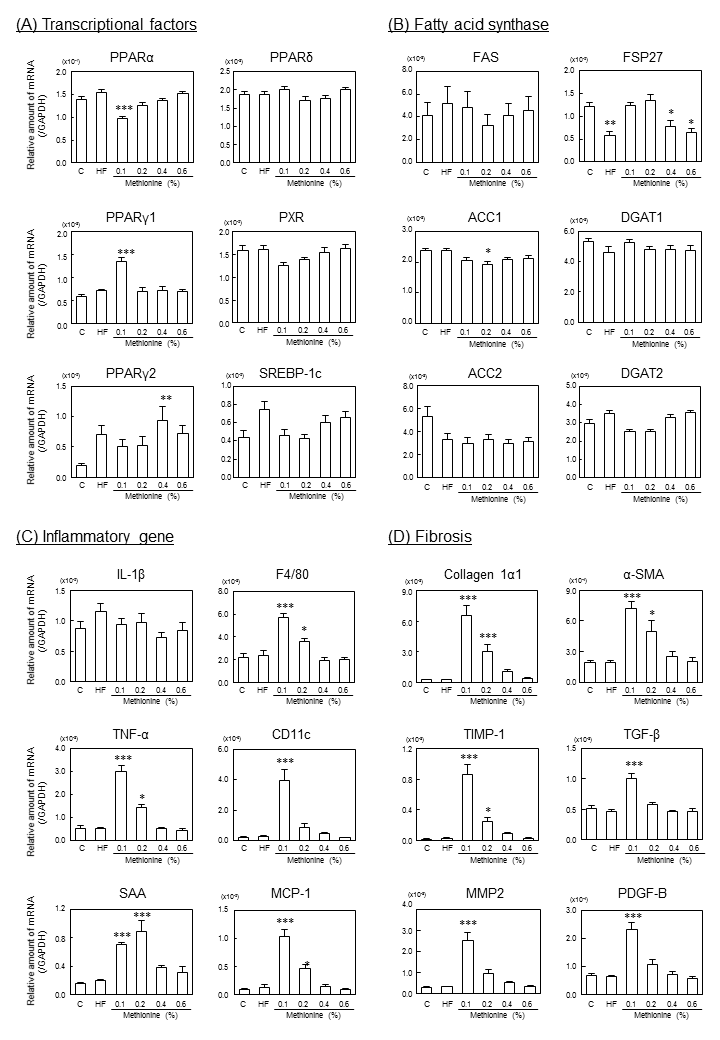

Supplement: S3 Fig — C57BL/6J mice (male, 10 weeks of age) were fed each experimental diet for 8 weeks. Total RNA was extracted from the liver, and expression levels of (A) transcriptional factors, (B) fatty acid synthase-related genes, (C) inflammatory-related genes and (D) fibrosis-related genes were measured by real-time RT-PCR methods. Data are represented as mean and SEM, n = 7 or 8 in each diet. *P < 0.05, **P < 0.01, ***P < 0.001 vs. control by one-way ANOVA with Bonferroni’s post-hoc test. (TIF) [file pone.0164191.s003.tif]

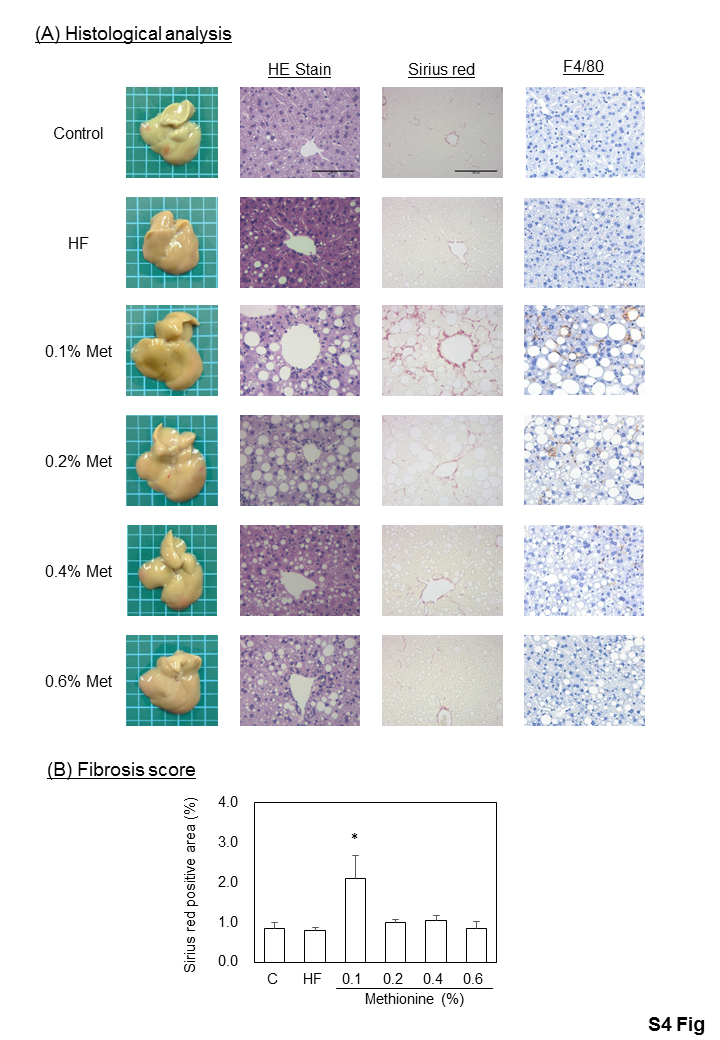

Supplement: S4 Fig — C57BL/6J mice (male, 10 weeks of age) were fed each experimental diet for 8 weeks. After overnight fasting, mice were killed and the liver was removed. (A) A pathological analysis with HE staining, serial red staining, and F4/80 immuno-staining was conducted. Bar indicates 100 μm. Original magnification, x200. (B) Fibrosis score was determined as the ratio of sirius red positive area to the whole area in each section. Data are represented as means and SEM, n = 7 or 8 in each diet. *P < 0.05 vs. control by one-way ANOVA with Bonferroni’s post-hoc test. (TIF) [file pone.0164191.s004.tif]
