# Supplementary material for: Evaluation of Methionine Content in a High-Fat and Choline-Deficient Diet on Body Weight Gain and the Development of Non-Alcoholic Steatohepatitis in Mice
Source: PLoS One. 2016 Oct 10;11(10):e0164191. doi: 10.1371/journal.pone.0164191 (PMC5056759; doi:10.1371/journal.pone.0164191)
Supplement: S1 Table — (DOCX) [file pone.0164191.s005.docx]

**S1 Table. Composition of Experimental Diets Used in This Study.**

|  | **Control** | **HF** | **HFCD + 0.1%** | **HFCD + 0.2%** | **HFCD + 0.4%** | **HFCD + 0.6%** |
| --- | --- | --- | --- | --- | --- | --- |
| Res. Diets Inc. ID | A06071314 | A06071304 | A06071309 | A06071318 | A06071319 | A06071317 |
| Ingredient (g) |  |  |  |  |  |  |
| L-Cystine | 4.2 | 4.2 | 4.2 | 4.2 | 4.2 | 4.2 |
| L-Isoleucine | 7.6 | 7.6 | 7.6 | 7.6 | 7.6 | 7.6 |
| L-Leucine | 15.8 | 15.8 | 15.8 | 15.8 | 15.8 | 15.8 |
| L-Lysine | 13.2 | 13.2 | 13.2 | 13.2 | 13.2 | 13.2 |
| L-Methionine | 5.1 | 5.1 | 0.8 | 1.7 | 3.4 | 5.1 |
| L-Phenylalanine | 8.4 | 8.4 | 8.4 | 8.4 | 8.4 | 8.4 |
| L-Threonine | 7.2 | 7.2 | 7.2 | 7.2 | 7.2 | 7.2 |
| L-Tryptophan | 2.1 | 2.1 | 2.1 | 2.1 | 2.1 | 2.1 |
| L-Valine | 9.3 | 9.3 | 9.3 | 9.3 | 9.3 | 9.3 |
| L-Histidine | 4.6 | 4.6 | 4.6 | 4.6 | 4.6 | 4.6 |
| L-Alanine | 5.1 | 5.1 | 5.1 | 5.1 | 5.1 | 5.1 |
| L-Arginine | 6 | 6 | 6 | 6 | 6 | 6 |
| L-Aspartic Acid | 12.1 | 12.1 | 12.1 | 12.1 | 12.1 | 12.1 |
| L-Glutamic Acid | 38.2 | 38.2 | 38.2 | 38.2 | 38.2 | 38.2 |
| Glycine | 3 | 3 | 3 | 3 | 3 | 3 |
| L-Proline | 17.8 | 17.8 | 17.8 | 17.8 | 17.8 | 17.8 |
| L-Serine | 10 | 10 | 10 | 10 | 10 | 10 |
| L-Tyrosine | 9.2 | 9.2 | 9.2 | 9.2 | 9.2 | 9.2 |
| Corn Starch | 502 | 72.8 | 77.1 | 76.2 | 74.5 | 72.8 |
| Maltodextrin 10 | 130.1 | 100 | 100 | 100 | 100 | 100 |
| Sucrose | 68.8 | 172.8 | 172.8 | 172.8 | 172.8 | 172.8 |
| Cellulose, BW200 | 50 | 50 | 50 | 50 | 50 | 50 |
| Soybean oil | 25 | 25 | 25 | 25 | 25 | 25 |
| Lard | 20 | 177.5 | 177.5 | 177.5 | 177.5 | 177.5 |
| Mineral Mix S10026 | 10 | 10 | 10 | 10 | 10 | 10 |
| DiCalcium Phosphate | 13 | 13 | 13 | 13 | 13 | 13 |
| Calcium Carbonate | 5.5 | 5.5 | 5.5 | 5.5 | 5.5 | 5.5 |
| Potassium Citrate, | 16.5 | 16.5 | 16.5 | 16.5 | 16.5 | 16.5 |
| Sodium BiCarbonate | 7.5 | 7.5 | 7.5 | 7.5 | 7.5 | 7.5 |
| Vitamin Mix V10001 | 10 | 10 | 10 | 10 | 10 | 10 |
| Choline Bitartrate | 2 | 2 | 0 | 0 | 0 | 0 |
| Total | 1039.35 | 841.55 | 839.55 | 839.55 | 839.55 | 839.55 |
| % (w/w) |  |  |  |  |  |  |
| Protein | 17 | 21 | 21 | 21 | 21 | 21 |
| Carbohydrate | 68 | 42 | 43 | 43 | 43 | 42 |
| Fat | 4 | 24 | 24 | 24 | 24 | 24 |
| kcal % |  |  |  |  |  |  |
| Protein | 18 | 18 | 18 | 18 | 18 | 18 |
| Carbohydrate | 72 | 36 | 36 | 36 | 36 | 36 |
| Fat | 10 | 46 | 46 | 46 | 46 | 46 |
