# Supplementary material for: Evaluation of Methionine Content in a High-Fat and Choline-Deficient Diet on Body Weight Gain and the Development of Non-Alcoholic Steatohepatitis in Mice
Source: PLoS One. 2016 Oct 10;11(10):e0164191. doi: 10.1371/journal.pone.0164191 (PMC5056759; doi:10.1371/journal.pone.0164191)
Supplement: S2 Table — (DOCX) [file pone.0164191.s006.docx]

**S2 Table. Primer Sequences (5’ - 3’) Used in This Study.**

GenBank ID Forward Reverse

PPARα BC016892 AAGACTACCTGCTACCGAAATG AACATTGGGCCGGTTAAGA

PPARδ MMU10375 GCCTCCATCGTCAACAAAGA TGATGTCACTGAAGGGCTTG

PPARγ1 U01664.1 GAGTGTGACGACAAGATTTG GGTGGGCCAGAATGGCATCT

PPARγ2 EF062476 TCTGGGAGATTCTCCTGTTGA GGTGGGCCAGAATGGCATCT

PXR AF031814 AGAGATCATCCCTCTTCTGCCAC GATCTGGTCCTCAATAGGCAGGT

SREBP-1c AB017337 CATCGACTACATCCGCTTCTT CACCAGGTCCTTCAGTGATTT

FAS NM_007988 GCTGCGGAAACTTCAGGAAAT AGAGACGTGTCACTCCTGGACTT

ACC1 AY451393 ACATTCCGAGCAAGGGATAAG GGGATGGCAGTAAGGTCAAA

ACC2 AY451394 CTTGTTGCCCAAGAGAGAGAA GGCTCCAAGTGGCGATAAA

FSP27 BC099676 GCTGAACCCTCAGGACTTTATT CTTGTAGCAGTGCAGGTCATAG

DGAT1 NM_010046 CCAACCATCTGATCTGGCTTAT GACTCAGCATTCCACCAATCT

DGAT2 NM_026384 AGTGGCAATGCTATCATCATCGT AAGGAATAAGTGGGAACCAGATCA

IL-1β M15131 TGGAGAGTGTGGATCCCAAGCAAT TGTCCTGACCACTGTTGTTTCCCA

TNF-α M13049 TCTCATGCACCACCATCAAGGACT TGACCACTCTCCCTTTGCAGAACT

SAA M11131 AGAGGACATGAGGACACCATTGCT AGGACGCTCAGTATTTGTCAGGCA

F4/80 X93328 TCAAATGGATCCAGAAGGCTCCCA TGCACTGCTTGGCATTGCTGTATC

CD11c BC057200 CCAATTGCTTCAACTCCCTAATG CATGGCTAAGGTATCACCTACAC

MCP-1 NM_011333 TCACCTGCTGCTACTCATTCACCA TACAGCTTCTTTGGGACACCTGCT

Collagen1α1 NM_007742 AGACCTGTGTGTTCCCTACT GAATCCATCGGTCATGCTCTC

TIMP-1 AY622853 GCACAGTGTTTCCCTGTTTATC AGTGCACAAGCCTAGATTCC

MMP2 NM_008610 GCTCTGTCCTCCTCTGTAGTTA GGTACAGTCAGCACCTTTCTT

α-SMA X13297 GACTCTCTTCCAGCCATCTTTC GACAGGACGTTGTTAGCATAGA

TGF-β M13177 CGAAGCGGACTACTATGCTAAA TCCCGAATGTCTGACGTATTG

PDGF-B AF162784 CTGAGCTGGACTTGAACATGA GTCTTGCACTCGGCGATTA

GAPDH M32599 TGATGCTGGTGCTGAGTATGTCGT TCTCGTGGTTCACACCCATCACAA
